# Development of a Surface Plasmon Resonance and Fluorescence Imaging System for Biochemical Sensing

**DOI:** 10.3390/mi10070442

**Published:** 2019-07-01

**Authors:** Lulu Zhang, Guijun Miao, Jing Zhang, Luyao Liu, Shisong Gong, Yichen Li, Dafu Cui, Yuanchen Wei, Duli Yu, Xianbo Qiu, Xing Chen

**Affiliations:** 1 College of Information Science and Technology, Beijing University of Chemical Technology, Beijing 100029, China; 2 State Key Laboratory of Transducer Technology, Institute of Electronics, Chinese Academy of Sciences, Beijing 100190, China

**Keywords:** surface plasmon resonance (SPR), fluorescence, combination, biosensor, cell

## Abstract

Surface plasmon resonance (SPR) biosensors are an extremely sensitive optical technique used to detect the changes in refractive index occurring at the sensor interface. Fluorescence involves the emission of light by a substance that has absorbed light or other electromagnetic radiation, and the parameters of the absorbed and emitted radiation are used to identify the presence and the amount of specific molecules in a specimen. SPR biosensors and fluorescence analysis are both effective methods for real-time detection. The combination of these technologies would improve the quantitative detection sensitivity of fluorescence analysis and the specificity of SPR detection. We designed and developed an SPR and fluorescence synchronous detection system. The SPR module was based on two kinds of modulation methods, and the fluorescence module was capable of switching between four wavelengths. The fluorescence microspheres and A549 cells of different concentration were both detected by the SPR and fluorescence method synchronously in real time. The fluorescent signal and the optical signal of the SPR were shown to correlate. The correlation coefficient for fluorescent microspheres detection reached up to 0.9866. The system could be used in cell analysis and molecule diagnosis in the future.

## 1. Introduction

Currently, there is a growing demand for the detection of biochemical substances. High-performance liquid chromatography (HPLC) and mass spectrometry (MS) are highly sensitive and selective methods that could detect different biochemical species in biological samples [1]. However, it requires complex sample preparation and time consuming procedures. In addition, the methods need specific expertise as well as well-trained operators. Enzyme-linked immunosorbent assays (ELISA) as an immunoassay method have been proven to be sensitive, specific, and inexpensive tools for detecting targeted analytes [2]. However, ELISA involves multiple washing steps and long reaction times.

Surface plasmon resonance (SPR) is a real-time, dynamic, label-free, and highly sensitive quantitative detection method developed in recent years [3,4,5,6,7]. It is based on the principle of physical optics, and surface plasmon wave (SPW) is used as a probe to detect the change of optical parameters of sensing media. This method is especially suitable for studying the interaction between biological molecules, such as detecting antigen-antibody interactions [8,9,10,11], studying the hybridization and adsorption of DNA, identifying lipid bilayer membranes, etc. [12,13,14]. This method avoids the shortcomings of traditional methods such as labeling, complex operation, and long detection time, and is becoming a research hotspot in the field of molecular analysis [15,16]. As a label-free technology, SPR relies on detecting changes in the molecular weight of target substances [17]. However, it is difficult to detect small molecules using SPR detection, and it obtains complex information from the sample by detecting changes in the refractive index [18].

Fluorescence detection is a specific detection method that has been widely used in the field of modern biochemical research and disease diagnosis [19]. However, although fluorescence detection does verify the presence of specific molecules in a medium, it is not easy to realize quantitative features [20,21]. Moreover, combining SPR and fluorescence systems requires increased manufacturing costs and makes the operation complex. Nevertheless, the combination of the SPR method and fluorescence technology in a single synchronous detection system can achieve complementary advantages. SPR would enhance the sensitivity of fluorescence detection of low molecular weight analytes or biomolecules present in complex samples as well as increase the detection dynamic range [22]. The combination of SPR and fluorescence detection also has many applications. For example, it could analyze the interaction between DNA polymerase, labelled nucleotides, and molecules attached to a substrate [23].

In 1991, Attridge et al. first reported the detection of hCG by the fluorescence analysis method based on SPR [24]. In 2002, Roy et al. monitored a variety of components of the molecular adsorption process using simultaneous SPR and fluorescence information [25]. In 2013, Chabot et al. identified the molecular mechanisms in cellular processes using simultaneous surface plasmon and enhanced fluorescence response [26]. In 2014, Toda et al. developed a surface plasma enhanced fluorescence spectrum system for point-of-care testing (POCT) diagnosis [27]. However, with POCT diagnostic devices having a fixed angle detection mode, the detection range of refractive index is limited.

In this paper, an SPR and fluorescence synchronous detection system was developed. The system was based on two kinds of modulation methods which included angle scanning and intensity detection. Simultaneously, the system can also detect the fluorescence signal by switching light wavelength among DAPI, FITC, TX RED, and CY5. The fluorescence microspheres and A549 cells of different concentrations were both detected by the SPR and fluorescence synchronous system in real time. The fluorescent signal and the SPR optical signal have been proven to correlate, with the correlation coefficient for the detection of fluorescent microspheres here reaching up to 0.9866.

## 2. Materials and Methods

### 2.1. SPR Sensor Setup

**Figure 1:**
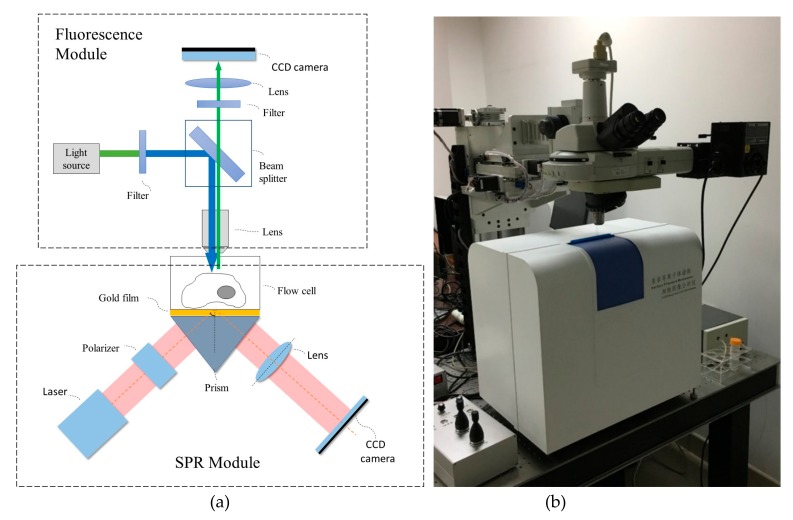
Optical structure (**a**) and photograph (**b**) of the surface plasmon resonance (SPR) and fluorescence synchronous detection system.

The SPR sensor was based on the prism coupling mode of the Kretschmann structure. As shown in Figure 1a, the proposed SPR sensor consisted of a red laser light source (635 nm peak wavelength), a prism with an equilateral triangle shape (n = 1.72; Beijing Glass Factory, Beijing, China), a SPR sensor chip, and a CCD camera (Basler, A102f, Ahrensburg, Germany). The laser, polarizing filter, and lens system were installed on a rotating arm. The CCD was mounted on another rotating arm, and both rotating arms were controlled by a stepper motor. 

### 2.2. Fluorescence Detection Device Setup and Characterization

The fluorescence detector was located on top of the SPR sensor. It contained a light source, filters, beam splitter, objective lens, imaging lens, and a CCD camera as a detector. As is common in fluorescence modules, the mercury lamp generated incident light which then passed through the filter and was reflected into the objective lens by the beam splitter. The fluorescence emitted by the fluorescent substance was then conducted to the CCD. Figure 1a shows the configuration design of the fluorescence detection device. The fluorescence system had the capability of switching between and detecting different optical signals, such as bright field, DAPI, FITC, TX RED, and CY5. The fluorescence image of a marker on the SPR chip of the system, under the excitation of four different light sources, was tested. A photograph of the SPR and fluorescence synchronous detection system is shown in Figure 1b.

### 2.3. Integration of SPR Chip and the Flow Cell 

**Figure 2:**
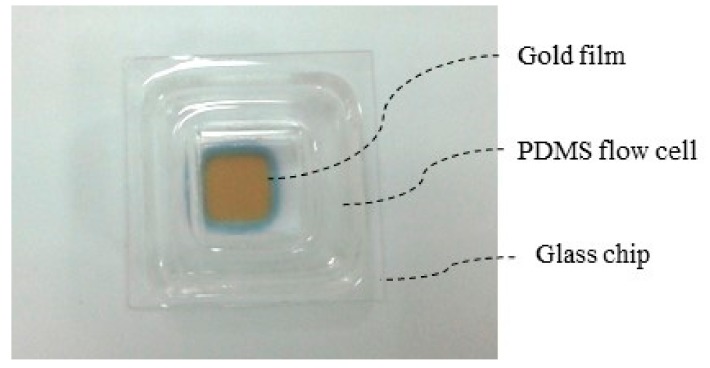
Photograph of integrated SPR chip and flow cell.

The SPR sensor chips were fabricated by magnetron sputtering technology on 20 × 20 × 0.17 mm glass slides, which were cleaned before experiment. We sputtered 2 nm chromium and 50 nm gold in an area of 5 × 5 mm. The flow cell, with a square shape, contained a mixture of PDMS oligomer and a crosslinking agent (Sylgard 184) with a 10:1 ratio. The mixture was degassed under vacuum, poured into a silanized glass mold, and then cured in an oven at 80 °C for 8 h. The square-shape PDMS flow cell was immobilized on top of the glass area to form a seal cavity for the various biochemical reactions. The SPR chip and the flow cell were integrated together as shown in Figure 2.

### 2.4. Preparation of Labeled Cell Samples

**Figure 3:**
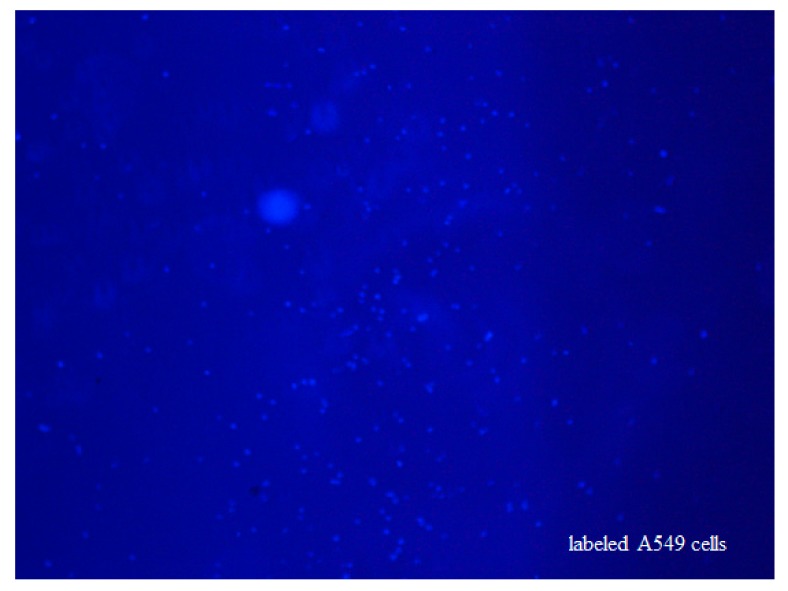
Fluorescence image of labeled A549 cells on a glass slide.

The non-small-cell lung cancer cell line of A549 was purchased from China Infrastructure of Cell Line Resources. Unless otherwise indicated, all cell-culture reagents were purchased from Life Technologies Corporation (Van Allen Way, Carlsbad, CA, USA). The A549 cells were cultured at 37 °C in 5% CO_2_ in a RPMI 1640 medium supplemented with 10% heat-inactivated fetal bovine serum, 100 units/mL penicillin, and 100 μg/mL streptomycin. Immediately prior to an experiment, cells were trypsinized, centrifuged, and resuspended in supplemented culture medium for the experiments. The A549 lung cancer cells were then fixed, labeled by Hoechst 33258, and placed in a centrifugal tube with a density of about 1E6/mL. A cell droplet of 30 μL was added to a glass slide, and the labeled fluorescence image was observed by the SPR-fluorescence system, as shown in Figure 3.

## 3. Results and Discussion

### 3.1. SPR Angular Scanning

**Figure 4:**
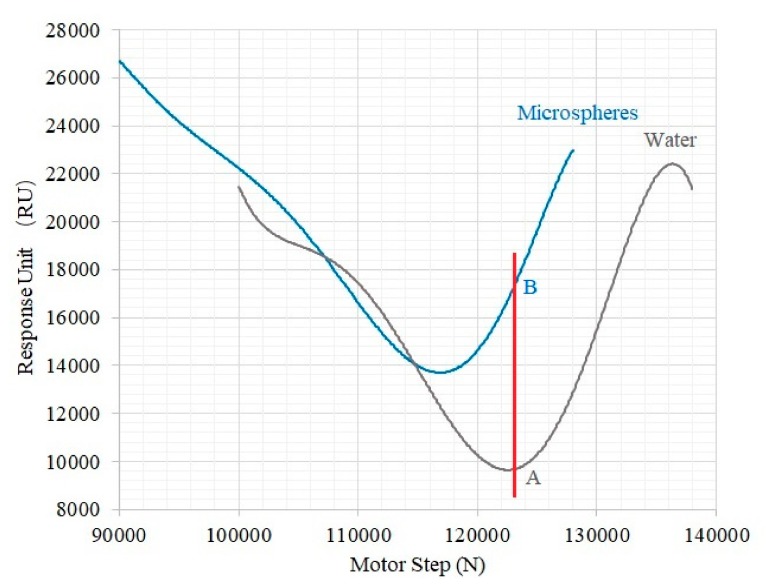
SPR peak curves of fluorescent microspheres and Mili-Q water by angular scanning.

During the SPR experiment, an SPR gold sensor chip was placed on top of the prism, with index matching oil added between the chip and the prism. A scan of the incident light angle was conducted in Milli-Q water to locate the absorption peak and the corresponding couple angle. Then, fluorescent microspheres were added to the surface of the SPR chip, and the incident light angle was changed via the motor and mechanical device. Figure 4 shows the experimental results of the SPR peak curves of fluorescent microspheres and Mili-Q water by angular scanning from 90,000 to 140,000 steps. In our system, we scan from a large angle to a small angle, so the larger the angle is, the smaller the motor step is.

### 3.2. Fluorescent Microspheres Detection

The 16 µm-diameter green fluorescent microspheres with an excitation wavelength of 495 nm and an emission wavelength of 519 nm were purchased from Tianjin BaseLine ChromTech Research Centre. The fluorescence image detection function and SPR detection function were tested on the integrated system. The fluorescent microspheres were diluted to 1000×, 100×, and 10× by adding deionized water. The diluted fluorescent microspheres were then added to a Petri dish, and the images were collected by the fluorescence system. The curves of SPR real-time monitoring in eight different areas were chosen. It was found that with an increase in the concentration of fluorescent microspheres to be measured, the number of fluorescent microspheres collected increases correspondingly.

**Figure 5:**
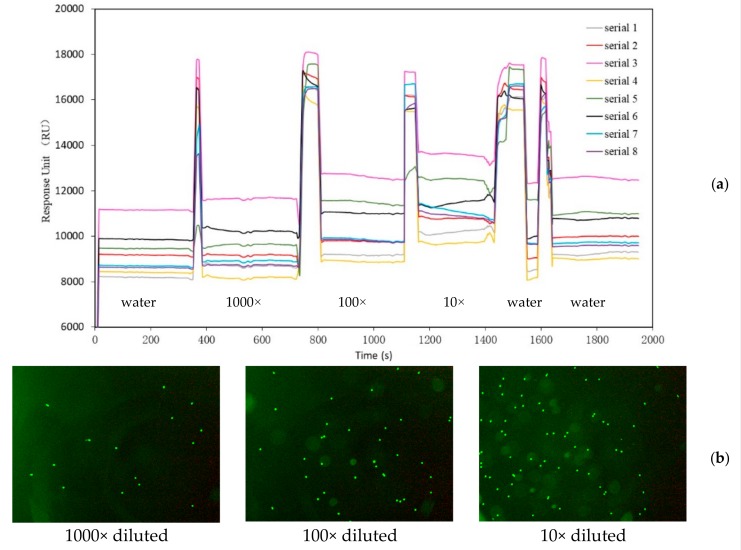
Monitoring of SPR signals (**a**) and fluorescence images (**b**) of fluorescent microspheres in real time with different concentrations.

Next, the synchronous detection of SPR and fluorescence signals was conducted. The incident angle was fixed at the resonant angle of deionized water to test different samples by the intensity detection method. The diluted fluorescent microspheres were added to the integrated SPR chip and the flow cell, which were placed on top of the prism initially. The test was conducted from a lower concentration to higher one. SPR and fluorescence images of fluorescent microspheres diluted at 1000×, 100×, and 10× were monitored in different regions. Eight regions were selected randomly on the SPR image, and the size of each region was the same. Because of the non-uniform spatial distribution of laser source, the initial intensities of different regions were also different, resulting in baseline differences. The curves of SPR real-time monitoring in eight different areas are shown in Figure 5a. It was found that with the increase of the concentration of fluorescent microspheres, the response unit (RU) of SPR detection increased accordingly. Finally, after the introduction of deionized water, the RU value decreased, but did not return to the initial signal value of deionized water.

In our system, the absorption peaks of different samples are different. For the same sample, the higher the concentration is, the higher the refractive index is, and the position of the absorption peak will move towards a large angle. In the case of fixed-angle detection, the increase of sample concentration corresponds to the increase of SPR detection signal, as shown in Figure 4 from point A to point B.

When an SPR signal was monitored in real time, the fluorescence images of microspheres were amplified five-fold and collected in real time. Figure 5b shows the fluorescence image of 1000×, 100×, and 10× diluted microspheres during SPR real-time detection. The fluorescence image illustrates that the number of fluorescent microspheres rises in a certain field as the concentration of fluorescent microspheres increases. However, the signal value of deionized water does not return to the initial value in the last cleaning process, as shown in Figure 5a. This phenomenon is related to the fact that some of the fluorescent microspheres still adhere to the surface of the SPR chip, which can be verified by fluorescent images after the cleaning process (figures not shown).

**Figure 6:**
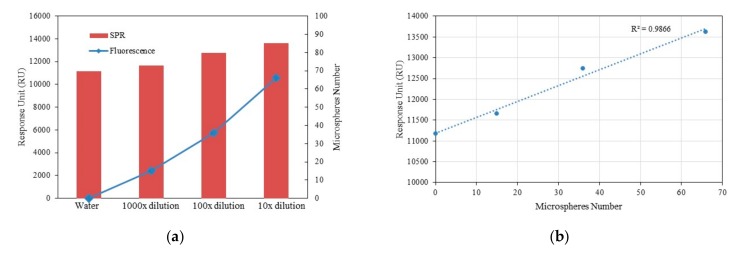
(**a**) The relationship between fluorescent signal (water, 1000×, 100×, and 10× diluted microspheres) and SPR detection signal (serial 1); (**b**) linear correlation coefficient between the whole fluorescence signal and SPR detection signal.

It was found that with an increase in the concentration of fluorescent microspheres, the number of fluorescent microspheres rises, and the optical signal of SPR increases commensurately. The relationship between the fluorescent signal (such as water, 1000×, 100×, and 10× diluted microspheres) and SPR detection signal (serial 1 as an example) is shown in Figure 6a. The correlation between the fluorescence signal and SPR detection signal is confirmed, and the two methods can complement each other, as a form of data validation. Figure 6b calculates the linear correlation coefficient between the whole fluorescence signal and SPR detection signal, and obtains R^2^ = 0.9866.

### 3.3. A549 Lung Cancer Cells Detection

**Figure 7:**
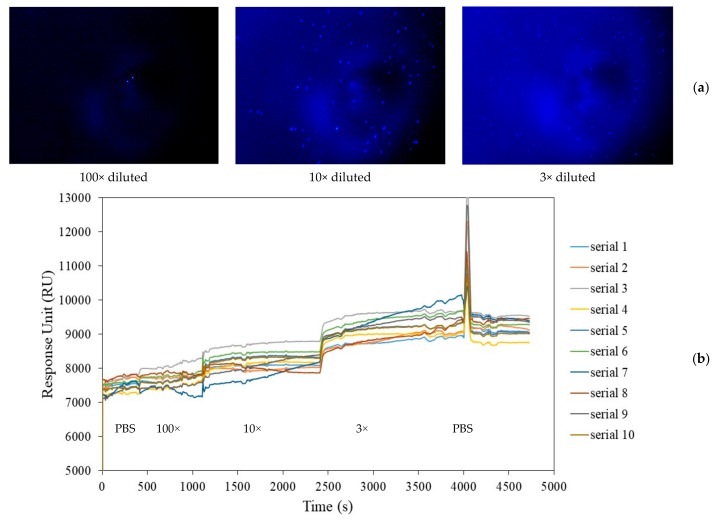
(**a**) Fluorescence images of A549 cell solutions with different concentrations (100×, 10×, and 3× diluted A549 cell solution) on an SPR chip; (**b**) curves of SPR real-time monitoring in ten different areas.

For A549 lung cancer cells detection, a new integrated SPR chip and flow cell with 198 µL PBS buffer was installed on the SPR-fluorescence synchronous detection system. By scanning the angle of incident light of the SPR device, the SPR resonance angle of PBS buffer was obtained, and the incident angle of the SPR was fixed near the resonance angle. It was difficult to clean the samples in the integrated flow cell due to the lack of inlet and outlet ports. As a result, A549 cell solutions of 2, 20, and 80 µL, with concentrations of approximately 1E6/mL cells, were introduced into the single-hole flow cell in sequence. Finally, the A549 cell solution concentration was diluted by 100×, 10×, and 3×, respectively, and the fluorescence images of the A549 cell solutions with different concentrations could be collected, as shown in Figure 7a. It was found that the number of cells observed increased significantly when 10× diluted A549 cell solution was added, while the number of cells with 3× diluted A549 cell solution correspondently did not change as obviously as that of 10× diluted A549 cell solution. The spike in Figure 7b at 4000 s is due to the introduction of air when changing the samples.

While collecting fluorescent images of an A549 cell solution, the changes in SPR signal in the A549 cell solution were monitored, as shown in Figure 7b. The curves of SPR real-time monitoring in ten different areas were chosen. It was found that with the increase in A549 cell solution concentration, the average RU value of SPR detection also increased. Finally, after injecting PBS buffer again, the RU value decreased, but did not return to the initial signal value of PBS buffer. This is related to the phenomenon that some A549 cells still adhere to the surface of SPR chip, these being similar to the fluorescent microspheres.

After the detection of microspheres or cells, the detection baseline did not return to the initial value because the microspheres or cells on the chip were not washed completely away, thus reducing the detection range of the refractive index. In the SPR fixed-angle detection process, the detection baseline increment was caused by the SPR peak movement. In this case, if the higher refractive index sample is detected again, the detection signal will much more easily reach saturation.

Compared with the fluorescent microspheres detection, the SPR signal for A549 cells detection was not very stable. The signal fluctuation of A549 was a little bit higher than the corresponding fluorescent microspheres signals. In addition, the variations in SPR signals in the different serials are also different. For example, in the detection of the 100× diluted cell solution, the SPR signal of serial 7 first increased and then decreased, while other serials ascended gradually. The SPR signals for serial 7 and serial 9 increased significantly in the detection process of the 10× diluted solution, but changes in the other serials were not particularly obvious. This indicates that the fluorescence of intracellular labeling is not as stable as that of fluorescent microspheres, so the signal fluctuates more greatly.

The SPR signal and fluorescence signal of A549 cells were also compared, and the phenomenon was different from that of fluorescent microspheres. The correlation coefficient between the SPR signal and fluorescence signal of fluorescent microspheres was 0.9866. For A549 cells, the SPR signals of the 3× and 10× diluted cell solutions changed obviously, and the ΔRU was more than 600, but the difference in fluorescence signals between them was not as great as that of the SPR signals.

Cells would secrete some protein molecules [28], which were probably adsorbed onto the bare gold film of the SPR system, resulting in non-specific adsorption. The effect of returning to baseline was worse in the cell experiments, so the correlation between SPR detection and fluorescence detection was also worse. Using the same method as the fluorescent microspheres experiment, the correlation coefficient between the SPR and fluorescent signals was calculated in the cell experiment as R^2^ = 0.8897, which was lower than that in the fluorescent microsphere experiment. In the future, the chemically modified SPR sensor chip could be used to reduce the non-specific adsorption phenomenon.

## 4. Conclusions

As identified by our measurements, we can conclude that as the concentration of the probing sample increases, so too do the fluorescent and optical signals of the SPR. Therefore, the overall fluorescent signal is correlated with the SPR detection signal. The two methods can complement and verify each other. Fluorescence detection is a specific detection method and can accurately locate the sample to be measured, but it is not easy to realize quantitative features. As a label-free technology, the SPR signal can obtain real-time information on the sample being measured and can analyze the kinetic characteristics of the reaction. Therefore, the combination of the SPR method and fluorescence technology can improve the quantitative detection sensitivity of fluorescence analysis and the specificity of SPR detection.

SPR biosensors and fluorescence analysis were combined into a real-time synchronous detection system. The SPR module was based on angle scanning and intensity modulation methods. The fluorescence module could switch between four different wavelengths. SPR real-time curves and fluorescence images of two kinds of samples were collected by the system. The fluorescent signal and the optical signal of the SPR were shown to correlate. The correlation coefficient for fluorescent microspheres detection reached up to 0.9866. The system could be used in cell analysis and molecule diagnosis in the future.
